# New CEACAM-targeting 2A3 single-domain antibody-based chimeric antigen receptor T-cells produce anticancer effects in vitro and in vivo

**DOI:** 10.1007/s00262-023-03602-4

**Published:** 2024-01-27

**Authors:** Iga Jancewicz, Magdalena Śmiech, Magdalena Winiarska, Radoslaw Zagozdzon, Pawel Wisniewski

**Affiliations:** 1 4Cell Therapies S.A., 59C Bojkowska Street, 44-100 Gliwice, Poland; 2 Department of Immunology, Medical University of Warsaw, 5 Nielubowicza St., Building F, 02-097 Warsaw, Poland; 3 Laboratory of Cellular and Genetic Therapies, Medical University of Warsaw, Banacha 1B, 02-097 Warsaw, Poland; 4 Department of Regenerative Medicine, The Maria Sklodowska-Curie National Research Institute of Oncology, 5 Roentgena Street, 02-781 Warsaw, Poland

**Keywords:** Nanobodies, Cancer microenvironment, Immune checkpoints, Cancer biomarker, CEACAM5/6, CAR T-cells

## Abstract

Recently, a breakthrough immunotherapeutic strategy of chimeric antigen receptor (CAR) T-cells has been introduced to hematooncology. However, to apply this novel treatment in solid cancers, one must identify suitable molecular targets in the tumors of choice. CEACAM family proteins are involved in the progression of a range of malignancies, including pancreatic and breast cancers, and pose attractive targets for anticancer therapies. In this work, we used a new CEACAM-targeted 2A3 single-domain antibody-based chimeric antigen receptor T-cells to evaluate their antitumor properties in vitro and in animal models. Originally, 2A3 antibody was reported to target CEACAM6 molecule; however, our in vitro co-incubation experiments showed activation and high cytotoxicity of 2A3-CAR T-cells against CEACAM5 and/or CEACAM6 high human cell lines, suggesting cross-reactivity of this antibody. Moreover, 2A3-CAR T-cells tested in vivo in the BxPC-3 xenograft model demonstrated high efficacy against pancreatic cancer xenografts in both early and late intervention treatment regimens. Our results for the first time show an enhanced targeting toward CEACAM5 and CEACAM6 molecules by the new 2A3 sdAb-based CAR T-cells. The results strongly support the further development of 2A3-CAR T-cells as a potential treatment strategy against CEACAM5/6-overexpressing cancers.

**Supplementary Information:**

The online version contains supplementary material available at 10.1007/s00262-023-03602-4.

## Introduction

Following successes in difficult-to-treat diseases such as malignant melanoma and lung cancer, checkpoint-targeted immunotherapy has been attempted in a range of solid cancers, including pancreatic ductal adenocarcinoma (PDAC) [1], refractory estrogen receptor (ER)-positive [2] and human epidermal growth factor receptor 2 (HER2)-positive breast cancers [2, 3]. Unfortunately, single-agent targeting of the classical immune checkpoints such as cytotoxic T-cell antigen 4 (CTLA-4) or the programmed death-receptor 1 (PD-1)/PD-L1 axis has produced little to no benefit for pancreatic cancer patients or endocrine-resistant breast cancer [2] and has been only moderately improved by combination approaches [1]. Nevertheless, multiple lines of evidence suggest that these malignancies occur in an immunosuppressive microenvironment [4], which is an obstacle to immunotherapeutic approaches [5]. Possibly, alternative immune checkpoints play an immunosuppressive role in these cancers. Among others, it has been suggested that members of the carcinoembryonic antigen-related cell adhesion molecule (CEACAM) family of proteins have immunoregulatory properties, making them potential targets for anticancer therapies. One potentially useful therapeutic agent, therefore, is the CEACAM-targeting 2A3 single-domain antibody (sdAb; also referred to as a V_H_H or nanobody) [6]. Previously, only CEACAM6 was described as the target of the 2A3 sdAb; however, the peptide sequence NRIGYSWYKG, identified as a potential epitope for the 2A3 sdAb [6], is highly similar between several members of the CEACAM protein family (please refer to Supp. Fig. S1). This implies a potentially broader spectrum of CEACAM proteins targeted by the 2A3 sdAb. We address this subject in the current work.

In addition to checkpoint inhibitors, a parallel line of anticancer immunotherapies are the adoptive approaches with the use of genetically modified immune effector cells. A prime example of such is T-cells expressing a chimeric antigen receptor capable of antibody-like recognition of the target plus T-cell receptor (TCR)-like activation and co-stimulation of the T-cell (CAR T-cells, reviewed in [7]). The most prominent breakthrough has been the use of CAR T-cell-based therapies to treat hematological malignancies, with several anti-CD19 and anti-B-cell maturation antigen (BCMA) strategies currently approved by the FDA. Following these successes in hematooncology, there have been multiple attempts to introduce CAR T-based strategies in the field of solid tumors [8]. One of the obstacles to such an approach is the scarcity of suitable cancer-associated targets. Another problem is that strong immunosuppression within some tumors may deactivate tumor-infiltrating CAR T-cells [9]. A solution for both of these problems might be to target the cancer-associated molecules that render the cancer cells resistant to cell-mediated cytotoxicity, i.e., the immune checkpoints. Indeed, PD-L1-targeting CAR T/NK-cells have recently been reported to be directly cytotoxic and also immunomodulatory in several solid tumor models in vitro and in vivo [10, 11]. However, the role of the PD-1/PD-L1 axis does not seem to play a dominant role in malignacies such as pancreatic cancer and ER-positive/HER2-positive mammary tumors. Instead, such a role could be proposed for CEACAM family molecules [12]. Therefore, in the current work, we present the investigation of the antitumor efficacy of T-cells bearing a novel CEACAM-targeted 2A3 sdAb-based CAR in PDAC or breast cancer models in vitro and in vivo.

## Materials and methods

### 2A3-CAR plasmid generation and lentivirus production

The 2A3 antibody [6, 13] sequence (Supplementary Fig. S2) along with CD28 and CD3ζ signaling domains was subcloned into a lentiviral plasmid with the CMV promoter (referred to as 2A3-CAR). For the in vitro experiments, lentiviruses were produced in 293FT cell line (Thermo Fisher Scientific). Cells were transfected with the pPACKH1 Lentivector Packaging mix (System Biosciences) and lentiviral vector according to the user manual using Lipofectamine2000 (Thermo Fisher Scientific) or NanoFect transfection reagent NF100 (Alstem*,* Richmond, CA, USA). The virus particles were collected 48 and 72 h after transfection using the Lenti-X Concentrator (TaKaRa, Kusatsu, Japan) according to manufacturer instructions or by centrifugation at 112,000 × *g* for 60 min at 4 °C (ProMab Biotechnologies, Inc. Richmond, CA, USA). The functional titers of the virus were determined by FACS analysis of CAR occurrence on cell membrane using anticamelid V_H_H Cocktail antibody (A02017, GeneScript, Piscataway Township, NJ, USA).

### Cell lines

BxPC-3 and Capan-1 cell lines were purchased from the ATCC (Manassas, VA, USA). MIA PaCa-2, MCF7, SK-BR-3 and MDA-MB-231 cell lines were in possession of 4Cell Therapies S.A. and were authenticated by Eurofins Genomics (Ebersberg, Germany). Capan-1 cells were cultured in IMDM, 20% FBS and 1× pen/strep (Biowest) on collagen I (Merck, Darmstadt, Germany)-coated plates. The BxPC-3 cell line was cultured with RPMI 1640 with 10% FBS and 1 × pen/strep (Biowest). The SK-BR-3 cell line was cultured in McCoy’s 5a supplemented with 20% FBS and 1 × pen/strep (Biowest). All other cell lines were cultured in DMEM high glucose supplemented with 10% FBS and 1 × pen/strep (Biowest).

### Stable cell line generation

MDA-MB-231 overexpressing CEACAM5 or CEACAM6 stable cell lines were generated using viral particles generated with the commercially available plasmids pLenti-GIII-CMV-CEACAM5 and pLenti-GIII-CMV-CEACAM6 (cat. No.: 15816061 and 15,817,061; ABM Goods, Richmond, Canada). Empty plasmid was used as a mock control. The CEACAM6 knockout MCF7 cell line was generated using a CRISPR/Cas9-based kit from Origene (cat. No.: KN402454; Rockville, MD, USA). Provided gRNA target sequences were: GTGAGCAGGACCTCCTTCCA and ACAGGAAAGTCACACTAAAC. Transfection of the MCF7 cell line was performed according to the manufacturer's protocol using Lipofectamine 2000. After transduction or transfection, respectively, cell lines were treated similarly as follows. Puromycin 2 µg/mL (POL-AURA, Poznań, Poland) was added to the culture medium 48 h after modification. Cells were diluted to obtain single colonies and then analyzed for the level of CEACAM5 and/or CEACAM6 protein by flow cytometry. At least two clones for each stable cell lines were cultured for further experiments.

### T-cell isolation, transduction and expansion

Peripheral blood mononuclear cells (PBMC) were isolated from human peripheral blood buffy coats obtained from the Regional Center for Blood Donation and Blood Treatment in Warsaw or from whole blood obtained in the Stanford Hospital Blood Center, Stanford according to an IRB-approved protocol (#13942). PBMC were isolated using Ficoll® Paque Plus (GE Healthcare, Chicago, IL, USA) according to the standard protocol. Then cells were suspended at 10^6^ cells/mL in AIM V Medium (Thermo Fisher Scientific), 10% FBS (Biowest) and 300 U/mL recombinant human IL-2 (rhIL-2; PeproTech, Cranbury, NJ, USA). Dynabeads™ Human T-Activator CD3/CD28 Kit (Thermo Fisher Scientific) was used for T-cell activation and expansion according to the manufacturer’s protocol. After 24 and 48 h, lentiviruses were added to the culture in 1 virion per cell ratio in the presence of 5 µg/mL DEAE-dextran (Merck). We used mock control T-cells transduced with lentivector coding scrambled sequence and unmodified control with untransduced T-cells. As the T-cells proliferated over the next 2–3 weeks, fresh medium supplemented with 300 U/mL rIL-2 was added to the culture every 2–3 days to maintain cell density at 1–3 × 10^6^ cells/mL.

### Co-culture

To evaluate the activity of 2A3-CAR T-cells to target cells, CAR T-cells were expanded for at least 10 days before use. Target cancer cell lines were seeded near confluency and cultured for 24 h. T-cells were then added to the tumor cell lines using an effector-to-target (E-to-T) ratio of 3:1 for pancreatic cancer cell lines, 5:1 for breast cancer cell lines and 10:1 for transduction-derived stable cell lines, as had been optimized earlier (data not shown). Cells were co-cultured in AIM V Medium (Thermo Fisher Scientific), 10% FBS and 1 × pen/strep (Biowest) for up to 48 h.

### *In vitro* cytotoxicity (RTCA method)

Cell viability was assessed using the real-time cell analyzer (RTCA; Agilent, Santa Clara, CA, USA). Co-culture was performed as described in the chapter *Co-culture* on 96-well E-plates (Agilent). Cells were co-cultured for 24 h. Each experiment was performed at least twice in technical duplicates.

### Cytokine secretion assay (ELISA method)

Media supernatants collected after co-culture were analyzed by ELISA for human IFN-γ levels using the Uncoated ELISA Kit from Thermo Fisher Scientific according to manufacturer’s instructions (Cat. no: 88-7316-88). Each experiment was performed at least three times in technical duplicates.

### Flow cytometry

Flow cytometry was used to measure CAR expression on T-cells, CAR T-cells degranulation after co-culture with tumor cells and CEACAM family protein levels on cell lines’ surfaces. Briefly, 0.5 × 10^6^ cells per test were washed, resuspended in 100 µL of FACS Staining buffer (0.5% BSA, 2 mM EDTA in 1 × PBS; Merck) and incubated with the appropriate antibody for 30 min at 4°C. After fixation in 1% paraformaldehyde (Merck) in PBS at room temperature for 15 min, cells were washed and resuspended in 200 µL of FACS Staining Buffer for analysis. Each probe was analyzed at least as technical duplicates. List of antibodies can be found in Supplementary Table 1.

### BxPC-3 xenograft tumor growth *in vivo*

All in vivo experiments were performed according to IACUC protocol (#SA-003). CIEA NOG (NOD.Cg-Prkdcscid Il2rgtm1Sug/JicTac) female mice, 5 weeks of age (obtained from Taconic Bioscience, Hudson, NY, USA) were used. Pancreatic BxPC-3 cancer cells (2 × 10^6^ cells/mouse) were injected subcutaneously into hind flanks on day 0. PBS (data not shown), unmodified T-cells or 2A3-CAR T-cells were injected (10^7^ cells/mouse; 5 mice per group) intravenously into mouse tail veins on days 1, 8 and 15 for the early intervention model. For the late intervention model, PBS (data not shown), T-cells or 2A3-CAR T-cells were injected when tumor volumes reached 100 mm^3^ (day 12) and on days 20 and 26. Tumor sizes were measured with calipers twice per week and tumor volumes were calculated using the following formula: (length × width^2^)/2. At the end of the study, surviving mice were killed and tumors were excised and photographed.

### Bioinformatic analysis

Protein BLAST analysis was performed using *blastp* default settings restricted to *Homo sapiens* species [14]. Amino acid sequence alignment was done using Clustal Omega [15] and analyzed using Jalview [16].

### Statistical analysis

Data were analyzed and plotted with GraphPad Prism 9 (San Diego, CA, USA). Comparisons between multiple groups were performed by one- or two-way ANOVA followed by Dunnett’s multiple comparisons test. Comparisons between two groups were made using *t*-test. The *p*-value < 0.05 was considered statistically significant.

## Results

### Generation of 2A3-CAR lentiviral vector and CAR expression in T-cells

**Fig. 1:**
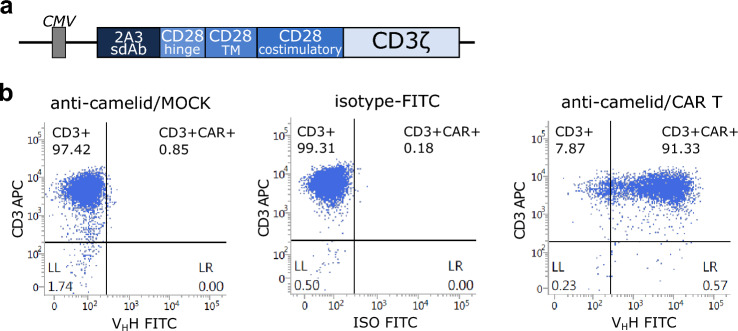
Expression of 2A3-CAR on transduced T-cells. **a** Modular diagram of the 2A3-CAR structure; **b** isotype control antibody on CAR expressing T-cells, 2A3-CAR detection on T-cells transduced with mock control lentivirus, 2A3-CAR detection on T-cells transduced with CAR lentivirus. Cells were stained with anti-CD3 [APC] (*y*-axis) and anticamelid VHH antibody [FITC] or isotype control (*x*-axis) to evaluate the percentage of cells expressing the 2A3 VHH antibody domain. Data are shown for one representative replicate. *sdAb*—single-domain antibody; *TM*—transmembrane domain

In this study, we have generated a novel chimeric antigen receptor comprising the 2A3 camelid sdAb along with CD28-CD3ζ signaling modules (Fig. 1a). As exemplified in Fig. 1b, CAR expression was detected in approx. 90% of the transduced T-cell population, independent of the buffy coat donor.

### Cytotoxicity and activation of 2A3-CAR T-cells *in vitro*

Six cancer cell lines were used to evaluate the interactions of 2A3-CAR T-cells with target cells: three pancreatic cancer cell lines (BxPC-3, Capan-1 and MIA PaCa-2) and three breast cancer cell lines (MCF7, SK-BR-3 and MDA-MB-231).

**Fig. 2:**
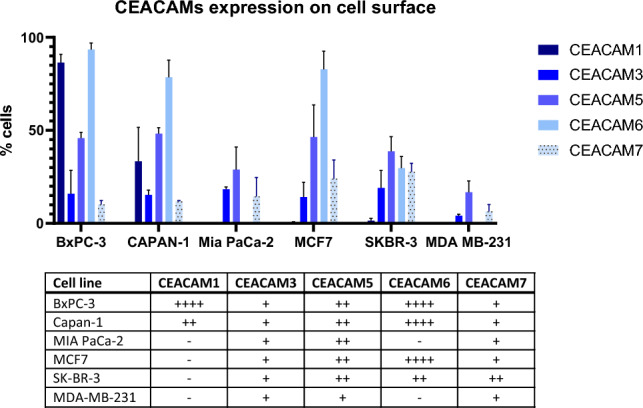
Flow cytometry analysis of CEACAM family protein abundance on cell surface. Analyzed cell lines differ in protein levels of analyzed CEACAM family proteins. The table presents unified data of protein abundance. Symbols of positiveness:−: < 3% positive cells, +: 4–25% positive cells, + +: 26–50% positive cells, +  +  +: 51–75% positive cells, +  +  +  +: 76–100% positive cells. Gating strategy is presented in Supplementary Fig. S3

Firstly, as shown in Fig. 2, we performed flow cytometric evaluation of the cell surface abundance of the CEACAM proteins with identified homology (Suppl. Fig. S1) to the published target epitope for 2A3 antibody. The analyzed cell lines differed in the pattern of CEACAM family protein abundance. CEACAM3, CEACAM5 and CEACAM7 were detected in all cell lines, however, with variable/low percentage of expression. Importantly, CEACAM6 was not detected on MIA PaCa-2 and MDA-MB-231 cell lines. CEACAM1 was the least abundant protein, present only on the surface of the BxPC-3 and Capan-1 cell lines.

**Fig. 3:**
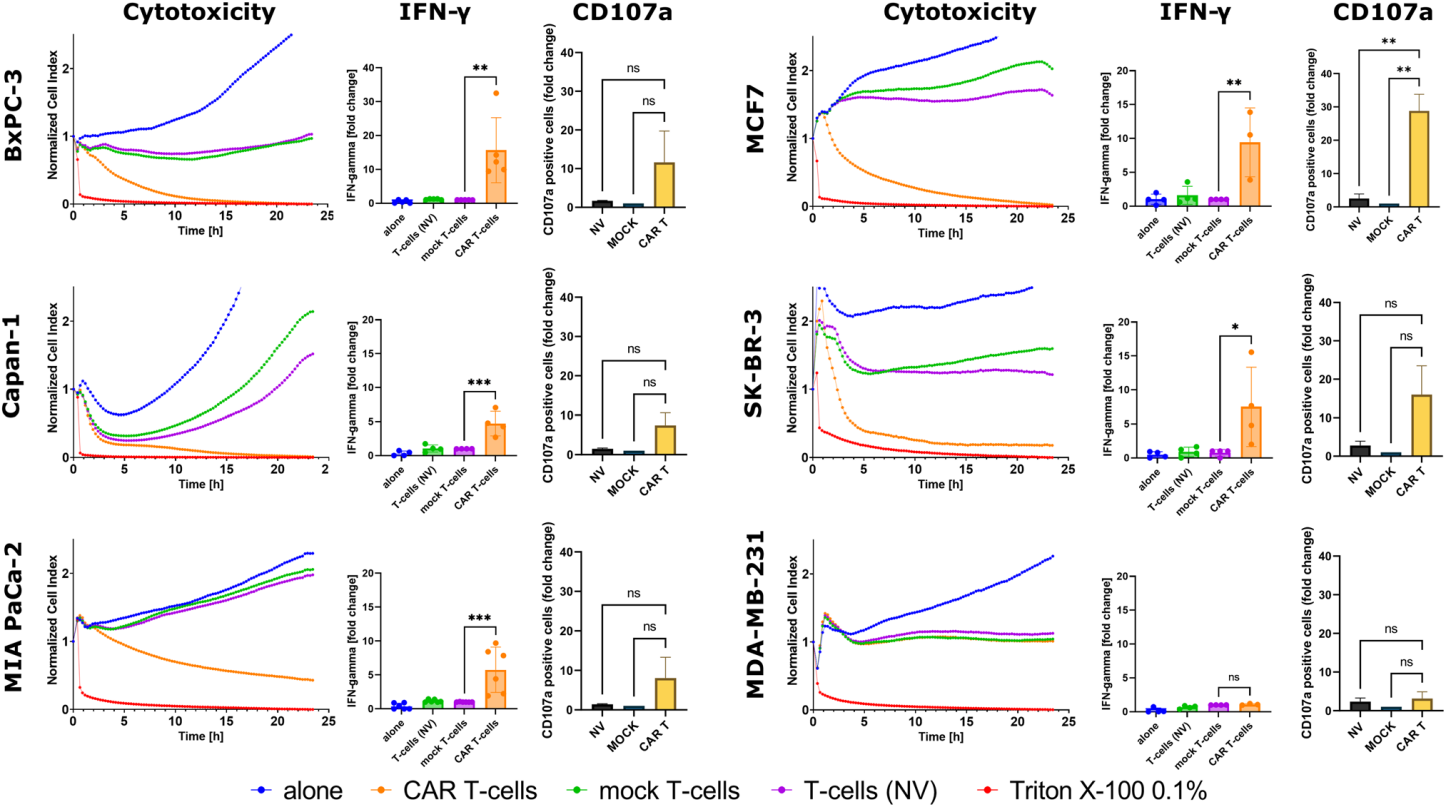
Cytotoxic activity, IFN-γ secretion and surface CD107a expression of 2A3-CAR T-cells against tumor cells in vitro. Cytotoxic effect observed in real time on RTCA instrument. O*range line*—anti-CEACAM6 CAR T-cells; *green line*—control mock T-cells; *purple linie*—untransduced T-cells; *blue line*—tumor cell line cells without T-cells; *red line*—cytotoxity positive control, cells treated with 0.1% Triton X-100. The RTCA experiment was repeated in duplicates four times. Representative data from one biological replicate are shown. Statistical analysis of RTCA experiment has been performed for the time point of 20 h after initiation of co-culture. *T*-test comparing CAR T-cells to other groups showed significant differences between CAR T-cells and all other groups, except MDA-MB-231 cell line. The data have been shown in Supplementary Table 2. IFN-γ levels in the supernatant collected after co-culture were determined by ELISA. Statistical analysis of ELISA and flow cytometry data was performed using two-way ANOVA followed by Dunnett’s multiple comparisons test. CD107a cell surface abundance was determined by Flow Cytometry and evaluated for statistical significance using ANOVA followed by Dunnett’s multiple comparisons test. Gating strategy is shown in Supplementary Figure S4. *NV*—untransduced T-cells, *mock*—mock control T-cells, *CAR T*—2A3-CAR T-cells. ***p < 0.001, **p < 0.01, *p < 0.05

Subsequently, cell lines were co-cultured with 2A3-CAR T-cells, mock T-cells or unmodified T-cells to assess the cytotoxic effects mediated by 2A3-CAR. We observed a potent cytotoxic effect of 2A3-CAR T-cells on almost all cell lines analyzed, with the exception of the MDA-MB-231 cell line. The data obtained from the ELISA for IFN-γ supported this observation. We observed significant increases in IFN-γ concentrations in the culture supernatants from cancer cells co-cultured with 2A3-CAR T-cells for almost all analyzed cell lines, except for MDA-MB-231 cell line (Fig. 3). No T-cell activation was observed when mock control T-cells, and unmodified T-cells were co-cultured with tumor cell lines.

We also tested degranulation of T-cells by assessing the CD107a levels on T-cell membranes after co-culture with the target cells. Again, co-culture of 2A3-CAR T-cells with almost all tumor cells, except for the MDA-MB-231 cell line, resulted in a trend toward increased CD107a expression on the T-cell surface (Fig. 3).

### Cytotoxicity of 2A3-CAR correlates with CEACAM5 and CEACAM6 abundance

Considering the fact that 2A3-CAR T-cells exerted cytotoxicity toward all but one of the cell lines studied, including the CEACAM6-negative MIA PaCa-2 cell line, we decided to reconsider the specificity of the 2A3-CAR. Based on the data from Baral et al. [6], we performed a BLAST analysis and sequence alignment to determine whether the epitope identified for the 2A3 antibody might be present in other proteins. The result suggested that seven (out of ten) consecutive C-terminal amino acids (*GYSWYKG*) in the proposed epitope sequence are identical in four CEACAM family proteins, i.e., CEACAM1, CEACAM3, CEACAM5 and CEACAM6 (Suppl. Fig. S1). For CEACAM7, six out of ten amino acids are identical with CEACAM6 in the sequence analyzed (Suppl. Fig. S1).

**Fig. 4:**
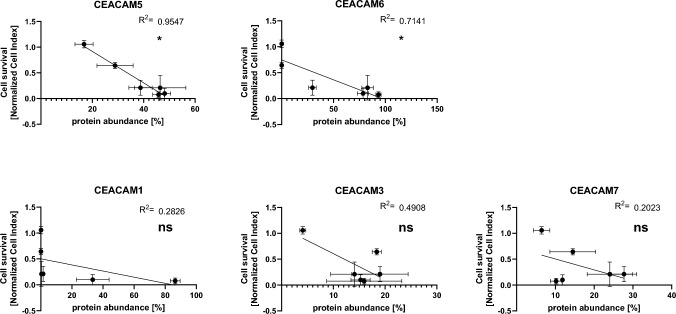
Correlation of CEACAM family protein abundance and cytotoxic effects against a panel of cell lines measured by RTCA method. We observed statistically significant correlations only for CEACAM5 and CEACAM6 proteins. Protein abundance is presented as the percentage of positive cells in flow cytometry experiment. Each point on one graph represents mean values of two experiments of flow cytometry and three experiments of RTCA for different cell line included in the study

Such high sequence homology between CEACAM family proteins suggests the similar cytotoxic effects of 2A3-CAR T against the mentioned family members of CEACAM. However, interestingly, the study shows that the cytotoxic effects of 2A3-CAR T-cells are statistically significantly correlated with cell surface abundance of CEACAM5 and CEACAM6 only (Fig. 4). Therefore, we decided to explore further the possibility that the 2A3 sdAb is cross-reactive (bi-specific).

### 2A3-CAR T-cells are cytotoxic against a MCF7 cell line derivative with CEACAM6 knockout

**Fig. 5:**
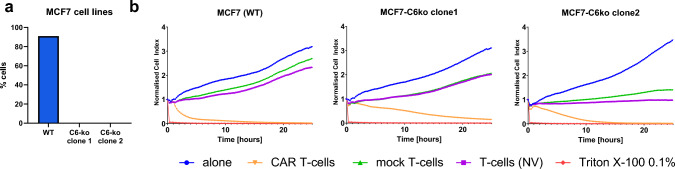
Cytotoxic effect of 2A3-CAR T-cells on a CEACAM6 knockout MCF7 cell line (MCF7-C6ko). **a** Flow cytometry data confirming knockout of CEACAM6 in the MCF7 cell line. **b** Cytotoxic effect observed in real time on RTCA instrument on MCF7 wild-type (WT) and knockout cell lines. O*range line*—anti-CEACAM6 CAR T-cells; *green line*—control mock T-cells; *purple line*—untransduced T-cells; *blue line*—tumor cell line cells without T-cells, *red line*—cytotoxity positive control (i.e., cells treated with 0.1% Triton X-100). The RTCA experiment was repeated in duplicates two times. Statistical analysis of RTCA experiment has been performed for the time point of 24 h after initiation of co-culture. *T*-test comparing CAR T-cells to other groups showed nonsignificant differences between CAR T-cells and all other groups. The data have been shown in Supplementary Table 3

To test the hypothesis that 2A3-CAR can recognize proteins other than CEACAM6, we generated a CEACAM6 knockout version of the MCF7 cell line (MCF7-C6ko) and performed the RTCA experiment with 2A3-CAR T-cells. Of note, one of the two CAECAM6-targeted sgRNAs used initially caused lethal effects each time; therefore, two clones obtained using only one sgRNA were used for the experiments. Conspicuously, 2A3-CAR T-cells exerted cytotoxic effects against the MCF7-C6ko cell line that were more potent than the effect of mock T-cells or untransduced T-cells (Fig. 5). However, the killing process of the MCF7-C6ko cell line was less dynamic than in the case of the MCF7 wild-type cell line. This suggests that activating 2A3-CAR T-cells does not depend on CEACAM6 alone; however, CEACAM6 is still a valid, albeit not unique, target for the 2A3-CAR.

### 2A3-CAR T-cells are cytotoxic against MDA-MB-231 cell line derivatives with overexpression of either CEACAM5 or CEACAM6 protein

**Fig. 6:**
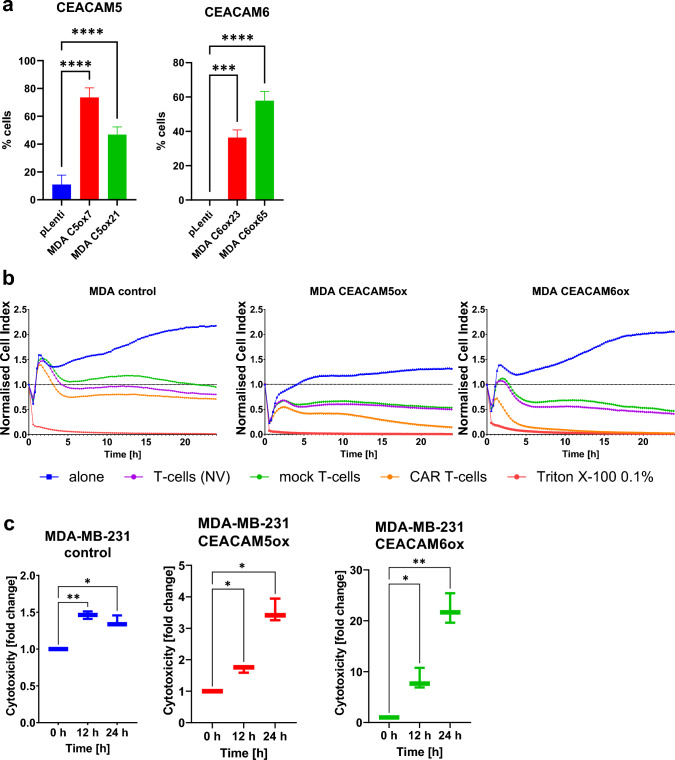
Cytotoxicity of 2A3-CAR T-cells against MDA-MB-231 cell line derivatives with CEACAM5 or CEACAM6 overexpression. **a** Flow cytometry data confirming overexpression of CEACAM5 or CEACAM6 in the MDA-MB-231 cell line. **b** Cytotoxic effect observed in real time on an RTCA instrument on MDA-MB-231 control and overexpressing cell lines. *Orange line*—2A3-CAR T-cells; *green line*—control mock T-cells; *purple line*—untransduced T-cells; *blue line*—tumor cell line cells without T-cells; *red line*—cytotoxity positive control (i.e., cells treated with 0.1% Triton X-100). The RTCA experiment was repeated in duplicates three times. Representative data from one biological replicate are shown. Statistical analysis of RTCA experiment has been performed for the time point of 24 h after initiation of co-culture. T-test comparing CAR T-cells to other groups showed significant differences between CAR T-cells and all other groups. The data have been shown in Supplementary Table 4. **c** Boxplots show the cytotoxic effect of 2A3-CAR T-cells on tumor cell lines as fold change compared to mock T-cells 0, 12 and 24 h post-start of the co-culture. Data from three repetitions in technical duplicates, analyzed by ANOVA followed by Dunnett’s post hoc test. ****p < 0.0001, ***p < 0.001, **p < 0.01, *p < 0.05

To further investigate the possibility of enhanced targeting by 2A3-CAR, we generated MDA-MB-231 cell line derivatives overexpressing either CEACAM5 or CEACAM6 proteins (Fig. 6a) and then analyzed the cytotoxicity of 2A3-CAR T-cells against these cell lines. We observed significant cytotoxic effects of 2A3-CAR T-cells against both MDA-MB-231 cell lines overexpressing either CEACAM5 (CEACAM5ox) or CEACAM6 (CEACAM6ox) proteins (Fig. 6b), as compared to mock T-cells or untransduced T-cells. In the case of the MDA-MB-231 control cell line, the cytotoxic effect was similar to that of the mock T-cells, suggesting that the effects of 2A3-CAR T-cells against CEACAM5ox and CEACAM6ox cell lines indeed depend on the overexpressed proteins. Importantly, the effect on MDA-MB-231 CEACAM6ox was approximately six times stronger than on MDA-MB-231 CEACAM5ox at the 12 and 24 h time points, even though levels of overexpression of CEACAM5 and CEACAM6 were similar (Fig. 6c). This again suggests that CEACAM6 should be considered a primary target for the 2A3 sdAb, and CEACAM5 may act as an auxiliary, but nevertheless also valid, target. All cytotoxicity experiments were performed on two clones of MDA-MB-231 overexpressing cell lines. Data from additional clones are presented in Supplementary Fig. S5.

### Antitumor efficacy of 2A3-CAR T-cells in the BxPC-3 xenograft model

**Fig. 7:**
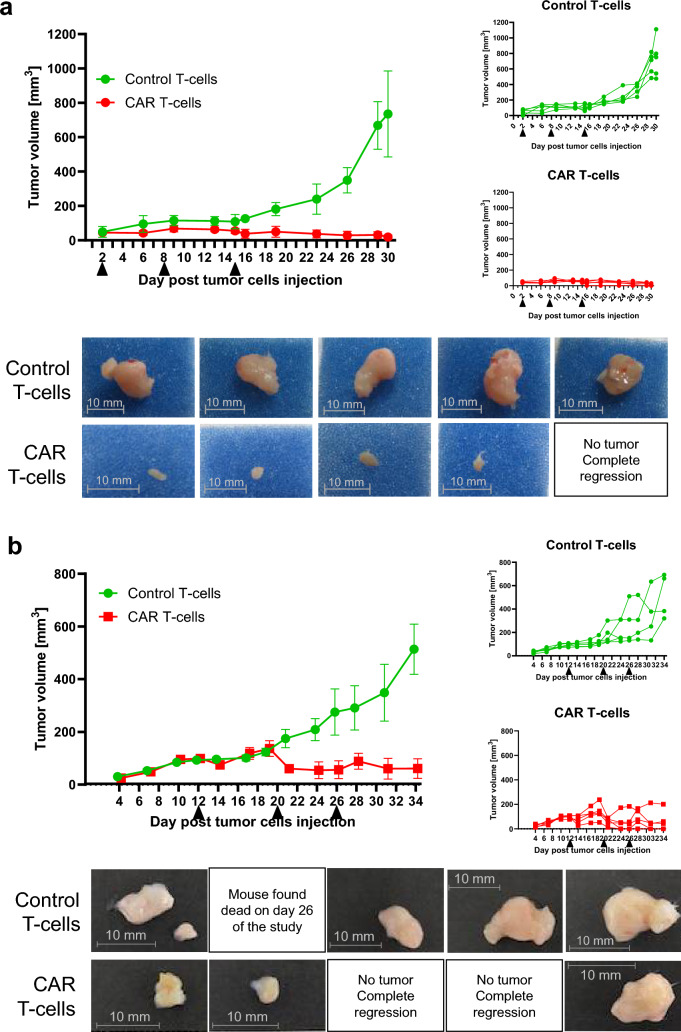
In vivo efficacy of 2A3-CAR T-cells in a human-to-mouse xenograft model. BxPC-3 cells were injected subcutaneously into the hind flank of CIEA NOG female mice. Mice were treated with control T-cells or 2A3-CAR T-cells intravenously into tail veins on days 1, 8 and 15 (*a*, early intervention model) or days 12, 20 and 26 (*b*, late intervention model) after tumor cell injections. (*Upper panels*) Mean tumor sizes ± SD and individual plots. (*Bottom panels*) Photographs of tumors at the end of the studies

Having established the efficacy of 2A3-CAR T-cells in vitro, we investigated the antitumor effects of these cells in the BxPC-3 xenograft model using both early and late intervention treatment regimens. As shown in Fig. 7a, treatment with 2A3-CAR T-cells significantly decreased the growth of all BxPC-3 xenografts (statistical analysis performed on day 30 data shows *p* < 0.0003 for control T-cells vs CAR T-cells) in early intervention regimen. In one mouse, a complete regression of tumor mass was observed (Fig. 7a). As early intervention treatment is not a realistic clinical scenario, a late intervention model was used. Injection of 2A3-CAR T-cells resulted either in control or regression of the tumors (statistical analysis performed on day 34 data shows *p* < 0.02 for control T-cells vs CAR T-cells; Fig. 7b). Also, in this part of the study complete regressions were observed in two of the CAR T-treated mice. Thus, 2A3-CAR T-cells showed high efficacy even against established tumors. No generalized toxicities were observed in the study (based on body weight measurements, data not shown). Altogether, the presented data clearly indicate the in vivo effectiveness of 2A3-CAR T-cells in a human pancreatic xenograft model.

## Discussion

The CEACAM family encoding genes are differentially expressed in numerous tissues and cell types in health and disease. Primarily, the role identified for CEACAMs is their involvement in intercellular adhesion and signal transduction [17]. However, it is currently well recognized that CEACAMs mediate complex biological functions and can play a significant role in cancer development and progression [18]. Among the CEACAM proteins, CEACAM5 is a well-known biomarker and indicator of recurrence in cancer patients and an established candidate for targeted experimental anticancer immunotherapies, e.g., using antibody–drug conjugates [19] or CAR T-cells [20].

In addition, CEACAM6 is abundantly overexpressed in a high proportion of cancers [21, 22]. It is a marker associated with cellular invasiveness and an unfavorable prognosis [23]. Deregulated expression of CEACAM6 directly affects the biology of cancer cells, e.g., by decreasing differentiation, inhibiting anoikis [24] or indirect increase in the metastatic potential [25]. Other consequences of aberrant CEACAM6 expression in cancer cells include increased proliferation and chemoresistance [22]. Importantly, high expression of CEACAM6 is also associated with immune suppression and low cytolytic T-cell activity in cancers [22]. This suggested that CEACAM6 may function as a negative immune checkpoint in cancer. Indeed, studies from colorectal cancer models have shown that CEACAM6 mediates inhibition of T-cell activation that is not redundant with the PD-1/PD-L1 axis [26]. A similar observation has also been reported in multiple myeloma, where binding and cross-linking of CEACAM6 by cytotoxic T-cells inhibited their activation and resulted in T-cell unresponsiveness [27]. Interestingly, in breast cancer, CEACAM6 status has been shown to be higher in both ER-positive tamoxifen-resistant breast cancer and HER-positive trastuzumab-resistant breast cancer than in treatment-responsive disease [21, 28], suggesting CEACAM6 as a potential target for the subsequent lines of therapy.

Because of its recognized role in cancer development and progression, CEACAM6 has become an attractive therapeutic or theragnostic target in a number of malignancies [13, 29, 30]. This line of investigation has been supported by in vitro observations that genetic downregulation of CEACAM6 results in decreased cellular invasiveness [23], reduction in the anoikis resistance and suppression of metastatic potential in cancer cells [31]. Following these reports, a number of anti-CEACAM6 antibodies, including BAY1834942 [26] and 2A3 [13], or antibody conjugates have been developed for clinical use. An interesting example of the latter is L-DOS47, in which a camelid anti-CEACAM6 antibody (AFAIKL2) is conjugated to urease [32]. Currently, a Phase 1/2 clinical trial is being conducted to evaluate the safety and tolerability of escalating doses of L-DOS47 in combination with doxorubicin, as well as preliminary antitumor activity in patients with previously treated advanced pancreatic cancer (ClinicalTrials.gov Identifier: NCT04203641). A Phase 1 clinical trial utilizing BAY1834942 in advanced solid tumors is also ongoing (NCT03596372). Interestingly, BAY1834942 when used in tumor cell/T-cell co-culture systems increased the production of T-cell cytokines and effector molecules (e.g., IFN-γ, TNFα, IL-2, granzyme B) and resulted in improved tumor cell killing, which indicates that this antibody may be a novel immune checkpoint inhibitor [26]. Nevertheless, the complexity of the immunosuppressive landscape in pancreatic and breast cancers raises questions about the efficacy of antibody-mediated targeting of CEACAM6 in these malignancies. This approach might not directly eliminate the cancer or cancer adjacent cells, as redundancy of other checkpoints could be expected. This would allow the tumor either to stay primarily resistant to the therapeutic strategy or to circumvent the blockade over time, as is most likely the case with antibody-mediated PD-1/PD-L1 checkpoint blockade. The solution may be the combination of CEACAM6 targeting with adoptive cytotoxic therapies, such as anti-CEACAM6 chimeric antigen receptor (CAR) T-cells [33]. CAR T-cells would physically eliminate CEACAM6-positive cells within the tumor burden. To validate this notion, hereby we applied this approach in the current study using the previously described anti-CEACAM6 2A3 llama single-domain antibody (sdAb, V_H_H) as a targeting domain in the new CAR presented.

Our work has demonstrated for the first time the enhanced targeting capabilities of the 2A3 sdAb, as we describe a cross-reactive targeting of primarily CEACAM6 molecule with an auxiliary targeting of a homologous protein, CEACAM5. Due to the high sequence homology, other CEACAM molecules, i.e., CEACAM1, CEACAM3 and CEACAM7, were also considered as potential targets for the 2A3-CAR T; however, our study did not confirm those assumptions. Nevertheless, the results of cross-reactive (i.e., CEACAM5/6) targeting of CEACAM family molecules by 2A3 sdAb raise hope for expanding the potential applications of 2A3-CAR T-cells against a wide range of tumors. Indeed, both CEACAM5 and CEACAM6 molecules have been reported to be highly overexpressed in multiple types of cancer [34]. This notion is further supported by the publications to date describing the generation and use of the NEO-201 antibody (IgG1 monoclonal antibody targeting variants of CEACAM5/6; reviewed in [35]). Of note, although both 2A3 and NEO-201 are CEACAM-targeting, the binding pattern seems to differ between these two antibodies, as NEO-201 does not bind SK-BR-3 cells [36], whereas we report significant interactions of 2A3-CAR T-cells with SK-BR-3 cell line (Fig. 3). The potential indications for the use of each of these antibodies will also differ, highlighting the novelty of the current report.

Notably, in the majority of cases the cross-reactivity of an CAR-mounted targeting domain is an undesired phenomenon. However, in the case for the 2A3 sdAb in our project, we do find beneficial the fact that this antibody targets an epitope conserved both in CEACAM6 and CEACAM5 proteins. This ensures more potent overall actions against CEACAM5/6 positive cells, even if one of these targets is missing on a percentage of cancer cells due to heterogeneity of the tumor. Obviously, this cross-reactivity potentially also increases the on-target, off-tumor toxicities of the 2A3 bearing CAR T-cell. This, however, can be alleviated by using, e.g., logical gating strategies (as reviewed in [37]) or intratumoral/loco-regional implantation of the 2A3-CAR T-cells. Notably, the latter strategy has already been safely attempted for anti-CEACAM5-CAR T-cells in clinical settings [38].

Additionally, 2A3 is an sdAb and is therefore expected to have significant physicochemical advantages over single-chain variable fragment (scFv) structures derived from antibodies such as NEO-201 for the purposes of CAR construction. These advantages include a reduced tendency to aggregate on the T-cell surface, which prevents premature T-cell activation and exhaustion, or the fact that the long CDR3 of sdAbs enables them to bind particular epitopes that are out of reach of conventional mAbs (reviewed in [39]). Therefore, our results substantiate further assessment of 2A3-CAR T-cells in the preclinical settings against CEACAM5/6-overexpressing cancers with parallel evaluation of the safety of such strategy, especially to address such issue as tissue penetration and bystander effect of 2A3-CAR T-cells or evaluate their effectiveness in the orthotopic settings.

An important issue to be taken into consideration during prospective preclinical safety tests of 2A3-CAR T-cells is the fact that rodents do not express a CEACAM6 (i.e., the most pronounced target for this CAR) analogue [40]. Therefore, testing CEACAM6-targeted therapies in regular mouse systems can only provide information on the efficacy of the given strategy, but should not be directly interpreted in terms of off-tumor, on-target toxicities. For this reason, the CEABAC transgenic mouse strain [41] or higher mammals, such as dogs or monkeys, must be used. The importance of this issue is underscored by the distribution pattern of CEACAM6 expression in healthy human tissues [42] such as lung, gastrointestinal tract or bone marrow. However, CEACAM6 expression in healthy tissues tends to be 1–2 log lower than expression in malignant cells [42], which decreases the overall risk for off-tumor toxicities. Should any significant toxicities be detected in non-rodent mammals, they can be potentially controlled, for example, by inducible expression of CAR, lower affinity CAR, or by using the transient expression electroporation method instead of the viral vector-based transduction of effector cells.

Another interesting aspect related to the targeting of CEACAM5/6 by CAR T-cells is the potential effects of this therapy on tumor-associated neutrophils, as these cells express high levels of CEACAM6 [43]. Indeed, some reports suggest that tumor-associated neutrophils that accumulate in, e.g., pancreatic cancers can regulate T-cell-dependent immunity [44] and promote metastasis [45]. It is, therefore, of great interest to determine whether 2A3-CAR T-cells can also target immunosuppressive elements of the cancer environment, such as neutrophils, in addition to the direct cytotoxic effect against malignant cells. Due to the lack of CEACAM6 in mice, the most appropriate experimental models may be the 3D co-culture in vitro models of tumor microenvironment [46] and humanized mouse models [47].

## Conclusions

In summary, our work contributes to the investigation toward establishing whether CEACAM-targeting 2A3-CAR T-cells can potentially be used as an effective immunotherapeutic agent against CEACAM5/6-expressing human malignancies, such as pancreatic or mammary cancers. In preclinical settings, our results indicate that camelid single-domain-based 2A3-CAR T-cells exert a potent direct cytotoxicity against human pancreatic and breast cancer cells. It remains to be investigated whether targeting CEACAM5/6 by CAR T-cells can provide an additional benefit to cancer therapy by acting on the constituents of the immunosuppressive elements of the tumor-associated microenvironment. It is also crucial to investigate potential on-target off-tumor cytotoxicity, as this is still the most important concern, regarding possible use of 2A3 sdAb-based CAR T-cells in humans. Our study suggests that the prospective clinical relevance of 2A3-CAR T-cell-based therapy in various malignancies including highly aggressive pancreatic cancer or breast cancer is worth further evaluation.

### Supplementary Information

Below is the link to the electronic supplementary material.

Supplementary file1 (PDF 1151 KB)

## Data Availability

Data are contained within the article or Supplementary Materials.
