# Correction to: New CEACAM-targeting 2A3 single-domain antibody-based chimeric antigen receptor T-cells produce anticancer effects in vitro and in vivo

**DOI:** 10.1007/s00262-024-03727-0

**Published:** 2024-07-05

**Authors:** Iga Jancewicz, Magdalena Śmiech, Magdalena Winiarska, Radoslaw Zagozdzon, Pawel Wisniewski

**Affiliations:** 1 4Cell Therapies S.A., 59C Bojkowska Street, 44-100 Gliwice, Poland; 2 Department of Immunology, Medical University of Warsaw, 5 Nielubowicza St., Building F, 02-097 Warsaw, Poland; 3 Laboratory of Cellular and Genetic Therapies, Medical University of Warsaw, Banacha 1B, 02-097 Warsaw, Poland; 4 Department of Regenerative Medicine, The Maria Sklodowska-Curie National Research Institute of Oncology, 5 Roentgena Street, 02-781 Warsaw, Poland; 5 Laboratory of Immunology, Mossakowski Medical Research Institute, Polish Academy of Sciences, 5 Pawinskiego Str., 02-106 Warsaw, Poland

## Correction to: Cancer Immunol Immunother. 2024 Jan 27;73(2):30. 10.1007/s00262-023-03602-4

In the original version of this article, the affiliation ‘’Laboratory of Immunology, Mossakowski Medical Research Institute Polish Academy of Sciences, 5 Pawinskiego Street, 02–106 Warsaw, Poland’’ for author Magdalena Winiarska was missing and e-mail address for author Magdalena Winiarska were incorrectly given as magdawiniarska@gmail.com but should have been mwiniarska@imdik.pan.pl.

