# Supplementary material for: New CEACAM-targeting 2A3 single-domain antibody-based chimeric antigen receptor T-cells produce anticancer effects in vitro and in vivo
Source: Cancer Immunol Immunother. 2024 Jan 27;73(2):30. doi: 10.1007/s00262-023-03602-4 (PMC10821984; doi:10.1007/s00262-023-03602-4)
Supplement: Supplementary file 1 — Supplementary file1 (PDF 1151 KB) [file 262_2023_3602_MOESM1_ESM.pdf]

Supplementary Tables

Supplementary Table 1

Supplementary Table 1. List of antibodies used for flow cytometry. Antibodies were purchased from Biolegend (SanDiego, CA, USA), GeneScript (Piscataway, NJ, USA), R&D Systems (Minneapolis, MN, USA), and Thermo Fisher Scientific (Waltham, MA, USA). Abbreviations: AF - alexa fluor, APC - allophycocyanin, FITC - fluorescein isothiocyanate, PE - phycoerythrin, n/a – not applicable.

Marker	Fluorochrome	Clone	Cat. no.	Source
CD3 (human)	APC	OKT3	317318	Biolegend
CD107a (LAMP-1) (human)	PE	eBioH4A3	12-1079-42	Thermo Fisher Scientific
CEACAM1 (human)	PE	283340	FAB2244P	R&D Systems
CEACAM3 (human)	Biotin	n/a	BAF4166	R&D Systems
CEACAM5 (human)	AF 488	487609	FAB41281G	R&D Systems
CEACAM5 (human)	AF 647	487609	FAB41281R	R&D Systems
CEACAM6 (human)	PE	KOR-SA3544	12-0667-42	Thermo Fisher Scientific
CEACAM7 (human)	AF 488	962703	FAB44781G	R&D Systems
camelid V_HH Cocktail	FITC	n/a	A02017	GeneScript
Streptavidin	FITC	n/a	11-4317-87	Thermo Fisher Scientific
rabbit IgG control	FITC	37C2	A02024	GeneScript
Mouse IgG1	AF 488	11711	IC002G	R&D Systems
Mouse IgG2A Isotype Control	AF 647	20102	IC003R	R&D Systems
mouse IgG1 kappa Isotype Control	PE	P3.6.2.8.1	12-4714-42	Thermo Fisher Scientific

Supplementary Table 2

Supplementary Table 2. Information from statistical analysis of RTCA experiment data for pancreatic and breast cancer cell lines. Analysis was performed for timepoint of 20 h after beginning the co-culture, using t-test according to Hanidi et. Al (2017) Bio Protoc. 7(24): e2646 (doi: 10.21769/BioProtoc.2646). All t-tests were done as paired one-tailed on groups of 4 reprtitions. *** p<0.001, ** p<0.01, * p<0.05

BxPC3				
CAR T-cells vs.	alone	T-cells (NV)	mock T-cells	Triton X-100
P value	0.0004	0.0113	0.0032	0.0154
P value summary	***	*	**	*
t, df	t=14.06, df=3	t=4.341, df=3	t=6.864, df=3	t=3.858, df=3
Capan-1				
CAR T-cells vs.	alone	T-cells (NV)	mock T-cells	Triton X-100
P value	0.002	0.0175	0.0185	0.0412
P value summary	**	*	*	*
t, df	t=7.989, df=3	t=3.673, df=3	t=3.593, df=3	t=2.572, df=3
MIA PaCa-2				
CAR T-cells vs.	alone	T-cells (NV)	mock T-cells	Triton X-100
P value	0.0176	0.0125	0.014	0.0253
P value summary	*	*	*	*

t, df	t=3.665, df=3	t=4.174, df=3	t=3.999, df=3	t=3.165, df=3
MCF-7				
CAR T-cells vs.	alone	T-cells (NV)	mock T-cells	Triton X-100
P value	0.0008	0.0001	0.0001	0.0363
P value summary	***	***	***	*
t, df	t=10.98, df=3	t=19.70, df=3	t=19.56, df=3	t=2.719, df=3
SK-BR-3				
CAR T-cells vs.	alone	T-cells (NV)	mock T-cells	Triton X-100
P value	0.0002	0.0088	0.0059	0.0106
P value summary	***	**	**	*
t, df	t=17.49, df=3	t=4.764, df=3	t=5.502, df=3	t=4.443, df=3
MDA-MB-231				
CAR T-cells vs.	alone	T-cells (NV)	mock T-cells	Triton X-100
P value	0.0145	0.4449	0.1306	0.0015
P value summary	*	ns	ns	**
t, df	t=3.952, df=3	t=0.1506, df=3	t=1.381, df=3	t=8.922, df=3

Supplementary Table 3

Supplementary Table 3. Information from statistical analysis of RTCA experiment data for MCF7 knock-out cell lines. Analysis was performed for timepoint of 24 h after beginning the co-culture, using t-test according to Hanidi et. Al (2017) Bio Protoc. 7(24): e2646 (doi: 10.21769/BioProtoc.2646). All t-tests were done as paired one-tailed on groups of 2 reprtitions.

MCF7 CEACAM5-knock-out				
CAR T-cells vs.	alone	T-cells (NV)	mock T-cells	Triton X-100
P value	0.105	0.1194	0.1803	0.2657
P value summary	ns	ns	ns	ns
Significantly different (P < 0.05)?	No	No	No	No
One- or two-tailed P value?	One-tailed	One-tailed	One-tailed	One-tailed
t, df	t=2.919, df=1	t=2.539, df=1	t=1.573, df=1	t=0.9060, df=1
Number of pairs	2	2	2	2
MCF7 CEACAM6-knock-out				
CAR T-cells vs.	alone	T-cells (NV)	mock T-cells	Triton X-100
P value	0.107	0.1801	0.1958	0.2637
P value summary	ns	ns	ns	ns
Significantly different (P < 0.05)?	No	No	No	No
One- or two-tailed P value?	One-tailed	One-tailed	One-tailed	One-tailed
t, df	t=2.861, df=1	t=1.575, df=1	t=1.415, df=1	t=0.9174, df=1
Number of pairs	2	2	2	2

Supplementary Table 4

Supplementary Table 4. Information from statistical analysis of RTCA experiment data for MDA-MB-231 cell lines with overexpression of CEACAM5 and CEACAM6 and control cell line. Analysis was performed for timepoint of 24 h after beginning the co-culture, using t-test according to Hanidi et. Al (2017) Bio Protoc. 7(24): e2646 (doi: 10.21769/BioProtoc.2646). All t-tests were done as paired, one-tailed on groups of 3 reprtitions. ** p<0.01, * p<0.05

MDA-MB-231 CEACAM5ox				
CAR T-cells vs.	alone	T-cells (NV)	mock T-cells	Triton X-100
P value	0.0039	0.0289	0.0333	0.0151
P value summary	**	*	*	*
t, df	t=11.19, df=2	t=3.978, df=2	t=3.676, df=2	t=5.618, df=2
MDA-MB-231 CEACAM6ox				
CAR T-cells vs.	alone	T-cells (NV)	mock T-cells	Triton X-100
P value	0.0155	0.0035	0.0127	0.0462
P value summary	*	**	*	*
t, df	t=5.538, df=2	t=11.88, df=2	t=6.161, df=2	t=3.057, df=2
MDA-MB-231 control				
CAR T-cells vs.	alone	T-cells (NV)	mock T-cells	Triton X-100
P value	0.0024	0.058	0.0313	0.0042
P value summary	**	ns	*	**
t, df	t=14.47, df=2	t=2.675, df=2	t=3.806, df=2	t=10.82, df=2

Supplementary Figures

Supplementary Figure S1

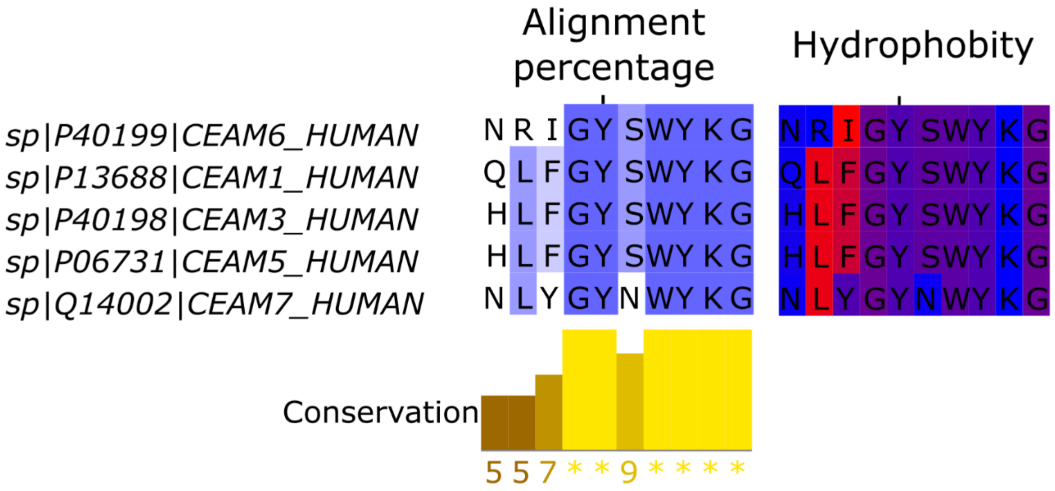

Supplementay Figure S1. Data from sequence alignment of the epitope sequence from CEACAM6, -1, -3, -5 and -7 presented in Jalview. Intensity of blue background of letters corresponds to the percentage of similarity of amino acids in the sequence. Hydrophobicity of amino acids is presented in red to blue scale, where red is most hydrophobic residue and blue is most hydrophilic residue.

Supplementary Figure S2

```
  1 QVKLEESGGG LVQAGGSLRL SCRTSGRTNS VYTMGWFRQA PGKEREFVAQ IMWGAGTNTH
 61 YADSVKGRFT ISRDSAESTV YLQMNSLKPE DTAVYYCAAN RGIPIAGRQY DYWGQGTQVT
121 VSS
```

Supplementary Figure S2. Aminoacid sequence of 2A3 sdAb, according to (Baral TN ey al. (2011) J Immunol Methods. 371: 70-80. doi: 10.1016/j.jim.2011.06.017).

Supplementary Figure S3

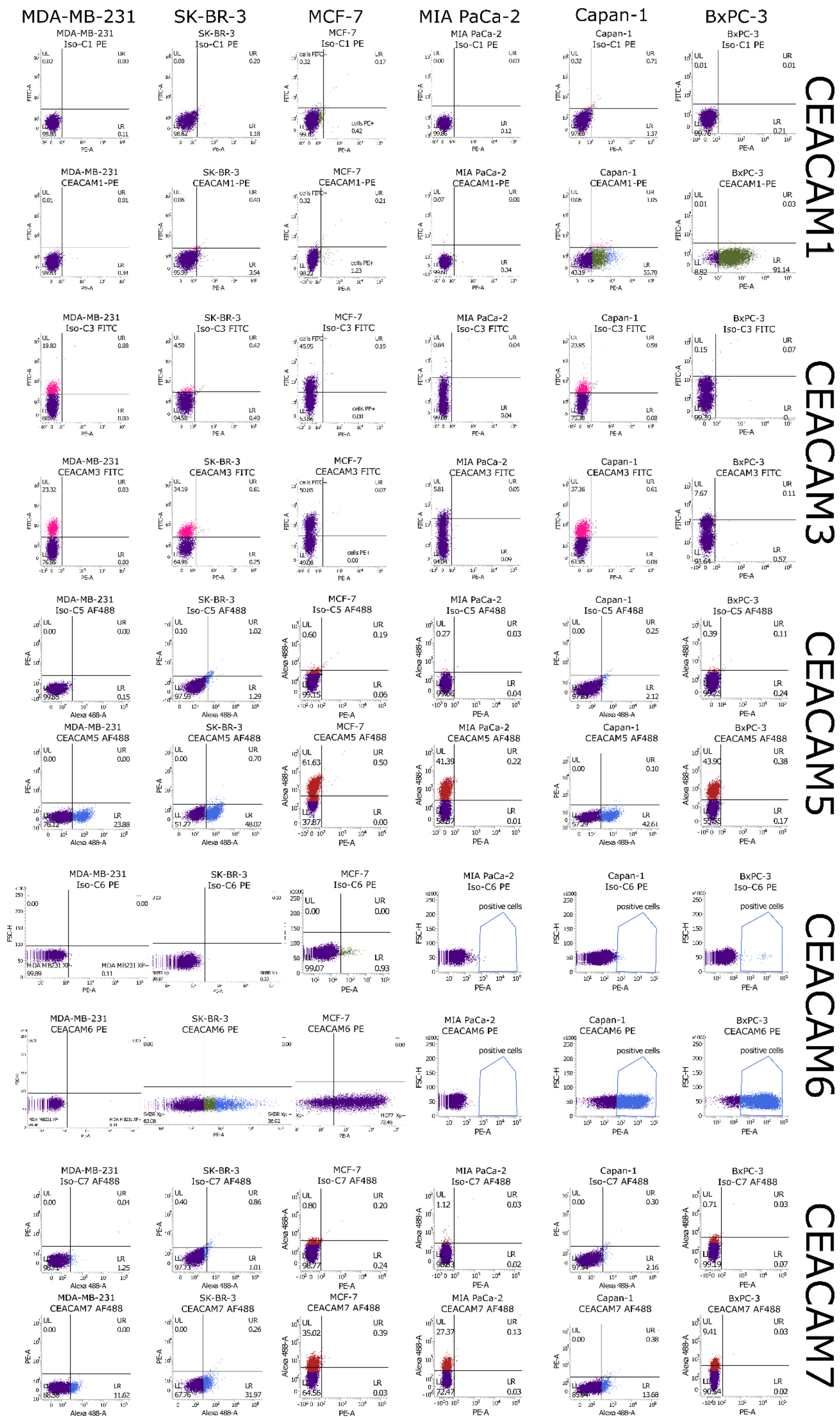

Supplementary Figure S3. The dot plots for the expression of each antigen by each cell line in regard to isotype control. Cell lines were stained with CEACAM1 (human) PE (283340, R&D Systems), CEACAM3 (human) Biotin (BAF4166, R&D Systems), Streptavidin FITC (11-4317-87, Thermo Fisher Scientific), CEACAM5 (human) AF488 (FAB41281G, R&D Systems), CEACAM6 (human) PE (12-0667-42, Thermo Fisher Scientific), CEACAM7 (human) AF488 (FAB44781G, R&D Systems). *Iso* – isotype control for the antobody.

Supplementary Figure S4

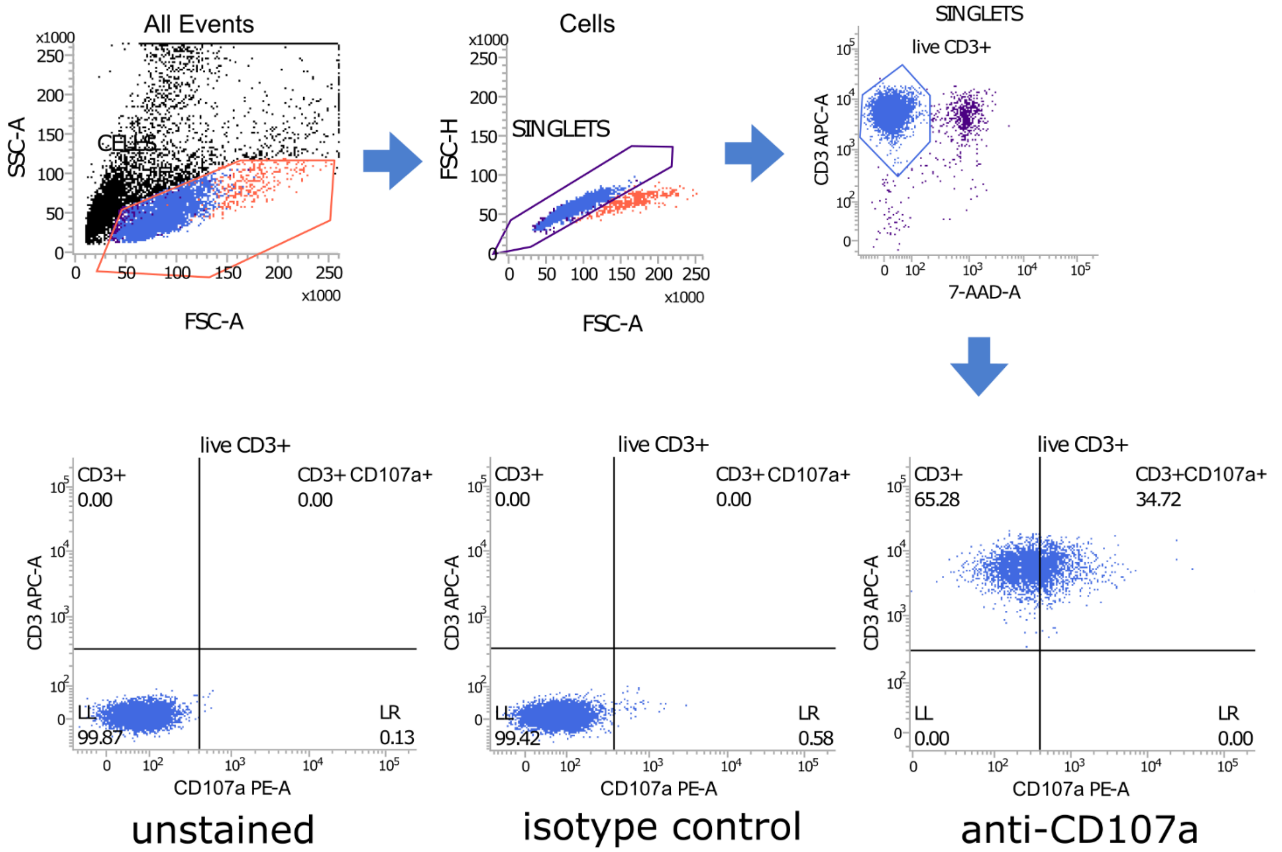

Supplementary Figure S4. Sequential gating to identify CD107a-positive lymphocytes. Staind with anti-CD3 APC (317318, Biolegend), anti-CD107a PE (12-1079-42, Thermo Fisher Scientific) and 7AA-D (420403, Biolegend).

Supplementary Figure S5

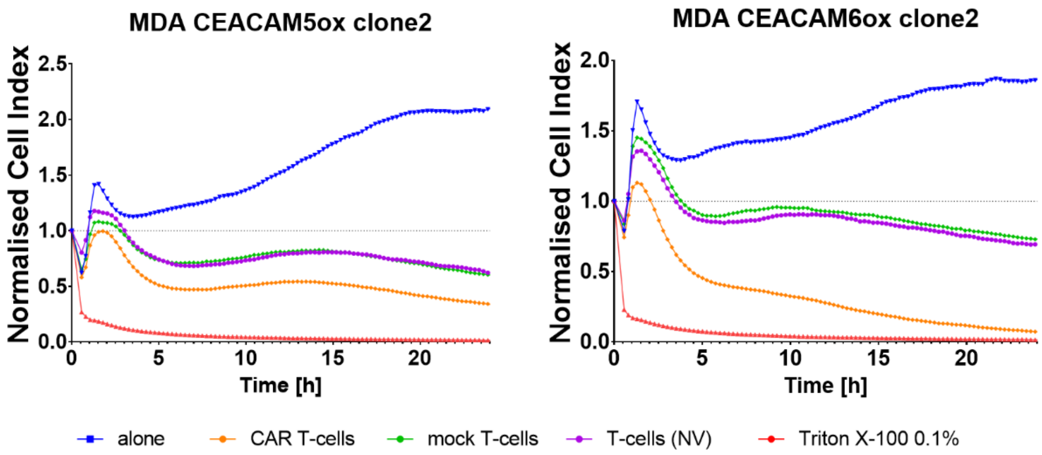

Supplementary Figure S5. Cytotoxicity of 2A3-CAR T-cells against MDA-MB-231 cell lines with CEACAM5 or CEACAM6 overexpression – data on additional cell line clones. *Orange line* - anti-2A3-CAR T-cells; *green line* - control mock T-cells; *purple linie* - untransduced T-cells (*purple line*); *blue line* – tumor cell line cells without T-cells, *red line* – cytotoxity positive control, cells treated with 0.1% Triton X-100.
